# Effect of the McGRATH MAC^®^ Video Laryngoscope on Hemodynamic Response during Tracheal Intubation: A Retrospective Study

**DOI:** 10.1371/journal.pone.0155566

**Published:** 2016-05-12

**Authors:** Masashi Yokose, Takahiro Mihara, Sayoko Kuwahara, Takahisa Goto

**Affiliations:** 1 Department of Anesthesiology, Kanagawa Children’s Medical Centre, Yokohama, Japan; 2 Department of Anesthesiology and Critical Care Medicine, Yokohama City University Graduate School of Medicine, Yokohama, Japan; University of Pennsylvania, UNITED STATES

## Abstract

**Background:**

Hypertension often occurs after tracheal intubation using a Macintosh laryngoscope and may lead to rare but serious complications. The Macintosh laryngoscope may increase the incidence of hypertension because it requires forced alignment of the oral and pharyngeal axes in order to view the glottis. In contrast, the McGRATH MAC video laryngoscope does not require this manipulation. The objective of this study was to evaluate the incidence of hypertension after tracheal intubation using a McGRATH laryngoscope compared with a Macintosh laryngoscope.

**Methods:**

Data of 360 consecutive patients who underwent general anesthesia with tracheal intubation by Macintosh laryngoscope or McGRATH video laryngoscope were obtained retrospectively. A total of 16 variables including patient characteristics, anesthetic drug used, and intubation techniques were extracted as potential factors affecting the incidence of hypertension after intubation. The incidence of hypertension after tracheal intubation was defined as an increase in systolic blood pressure (SBP) >20% of values immediately before intubation. Propensity scoring with inverse probability weighting was used to calculate the odds ratio for the incidence of hypertension after intubation with a McGRATH video laryngoscope as the primary outcome. The mean difference in SBP change between the two laryngoscopes was also calculated.

**Results:**

A McGRATH laryngoscope was used in 68 of 360 patients (18%). The numbers of patients who increase in systolic blood pressure of more than 20% was 189 patients (53%). The odds ratio for the use of a McGRATH laryngoscope was 0.43 (95% confidence interval (CI), 0.19–0.96; P = 0.04). The mean difference in SBP change between the two laryngoscopes was -8.6 mmHg (95% CI, -17.4 to 0.2; P = 0.06).

**Conclusions:**

The use of a McGRATH laryngoscope may reduce the incidence of hypertension after tracheal intubation compared to the Macintosh laryngoscope.

## Introduction

Hypertension often occurs after tracheal intubation in general anesthesia, and is affected by various factors such as patient characteristics [1–4], dose of opioid administered at induction [5, 6], and type of intubation device used [6–9]. Because it may lead to rare, but serious complications such as myocardial infarction or brain hemorrhage [10, 11], anesthesiologists should be careful to minimize the incidence of hypertension during tracheal intubation.

The McGRATH video laryngoscope (McGRATH MAC^®^, Aircraft Medical Ltd, Edinburgh, UK) is a novel intubating device. It has a handle and blade, which is similar to a Macintosh laryngoscope blade in shape, and a small camera and light source at the tip of the blade. The McGRATH video laryngoscope provides a clear image of the vocal cords and laryngeal tissue on the liquid crystal display screen attached to the end of the handle.

The Macintosh laryngoscope could be responsible for the high incidence of hypertension, because it requires forced alignment of the oral and pharyngeal axes in order to view the glottis. In contrast, the McGRATH video laryngoscope does not require this manipulation. Therefore, we hypothesized that a lower incidence of hypertension after tracheal intubation would be observed with a McGRATH laryngoscope compared to a Macintosh laryngoscope. The aim of this retrospective study was to investigate our hypothesis using the propensity score technique.

## Materials and Methods

This retrospective study was conducted according to the Declaration of Helsinki and was approved by the institutional ethical committee of the Yokohama City University Hospital, Yokohama, Japan (number, B141201002) on October 2014. Based on the opinion of our local ethical committee, written informed consent was not obtained from study participants. Data were obtained retrospectively from the medical and anesthetic records of 360 consecutive patients aged ≥18 years who underwent general anesthesia at the Yokohama City University Hospital (Yokohama, Japan) between July 1, 2014 and October 3, 2014. All patient data was anonymized and de-identified prior to analysis. Exclusion criteria included an awake intubation, rapid sequence induction, nasal intubation, use of a laryngeal mask, use of an intubation device other than a Macintosh or McGRATH laryngoscope, patients undergoing cardiovascular or thoracic surgery, and a diagnosis of pheochromocytoma. When repeated intubation attempts were evident or strongly suspected from the medical record (i.e., documentation of repeated intubation attempts, prolonged intubation [defined as >20 min from induction of anesthesia to intubation], ≥2 interruptions in the end-tidal carbon dioxide concentration recording), the patient was also excluded from analysis.

The following 16 variables were extracted as potential factors to affect the incidence of hypertension after tracheal intubation: (1) age; (2) sex; (3) body mass index; (4) systolic blood pressure (SBP) on admission; (5) history of hypertension; (6) antihypertensive therapy; (7) diabetes mellitus; (8) estimated glomerular filtration rate; (9) current smoking status; (10) American Society of Anesthesiologists Physical Status; (11) intubation operator; (12) intubation tube size; (13) time to intubation; and doses of (14) fentanyl, (15) propofol, and/or (16) remifentanil at induction. These variables were chosen based on previous studies [1–4, 12–14] and clinical knowledge. History of hypertension was defined as a diagnosis of hypertension prior to admission. Current smoking status was defined as a history of smoking within 1 month of surgery. The intubation operators were classified into the following 3 categories: junior anesthetic resident with <3 months anesthetic experience, senior anesthetic resident with <5 years working as an anesthesiologist, and staff anesthesiologist. The time to intubation was defined as the time between the start of anesthesia and completion of intubation. The start of anesthetic induction was defined as the time sedative drugs were first administrated according to the medical record. Completion of intubation was defined as the time the end-tidal carbon dioxide tension, which was recorded every minute in the anesthetic record, increased above zero after intubation. Patients with diabetes mellitus were defined as patients treated with oral diabetic drugs or insulin. In our institution, we usually place the arterial catheter after the intubation. Thus, we collected the data of noninvasive blood pressure. In most cases, the interval of blood pressure measurement was two to five minutes. Pre-SBP and post-SBP were defined as the SBP measured noninvasively immediately before and after intubation, respectively. The hemodynamic response was evaluated using the following calculation:

$$\%SBP\;change\;=\;\left(post-SBP\;-\;pre-SBP\right)\;pre-SBP^{-1}$$

It is generally accepted that, during anesthesia, the blood pressure should be maintained within 10–20% of the baseline level [15]. Patients with changes outside this range are at increased risk of complications [16]. Hypertensive emergencies are typically defined as acute elevations of blood pressure >20% of baseline [15]. Thus, hypertension was defined as an increase in SBP of more than 20% compared to the SBP immediately prior to intubation.

Assuming a 50% incidence of hypertension, a minimum of 320 patients were required to perform logistic regression analysis using 16 variables. We decided to increase the sample size by approximately 10% to allow for potential difficulties in data collection. Therefore, a total of 360 patients were enrolled. All parametric data are expressed as the mean and standard deviation or nonparametric data as the median and interquartile range (25th to 75th percentile). Data normality was tested using the Kolmogorov–Smirnov test. The patients included in our analysis were divided into 2 groups: those with use of the McGRATH laryngoscope and those with use of the Macintosh laryngoscope. The patient characteristics and pre-anesthetic values of each parameter were compared between these groups using the unpaired t-test, Mann–Whitney U-test, or Fisher's exact test, as appropriate. We reported both unadjusted and propensity technique adjusted p values for each parameter.

Because anesthetic management including the induction or airway management was at the discretion of the attending anesthesiologist, the intubation device was not randomly assigned. Therefore, patient characteristics were imbalanced between two groups, so we used a propensity score technique to account for potential confounding. We chose to use propensity score weighting, because it was the most appropriate method given the relatively small number of subjects [17]. The propensity score was defined as the probability of receiving the intervention (i.e., intubation with a McGRATH laryngoscope). We adopted an inverse probability weighted (IPW) approach, which uses the propensity score to create a weighted sample (“pseudopopulation”) in which all measured characteristics are balanced between the intervention groups, thereby removing confounding by these characteristics. The propensity score for McGRATH laryngoscope use was estimated using a logistic regression model in which the dependent factor was the intervention group and the independent factors were 16 potential predictors described above. All parameters were checked for multicollinearity. The discrimination and calibration of the model for estimating the propensity score were examined using the C-index and Hosmer-Lemeshow tests, respectively. Each patient was inversely weighted by the probability of that patient receiving the intervention they received. We assessed the effect of using a McGRATH laryngoscope in the weighted sample using a generalized linear model. Characteristics that remained imbalanced, which defined the adjusted p value >0.2, in the weighted sample were additionally included as independent variables in the generalized linear model to remove residual confounding by these parameters. The odds ratio and 95% confidence interval (CI) for the incidence of hypertension after intubation with a McGRATH video laryngoscope were calculated as the primary outcome. Propensity scoring with IPW was used for the following additional analyses: (1) the odds ratios for the incidence of hypertension when hypertension was defined a >20% increase in MBP or DBP, (2) the mean difference in the change of SBP, MBP, or DBP after intubation between the two laryngoscopes, (3) the mean difference in the time to blood pressure measurement or the end-tidal carbon dioxide (EtCO2) tension after intubation between the two laryngoscopes. For all analyses, P values <0.05 were considered statistically significant. All statistical analyses were performed using the R statistical software package, version 2.13.0 (R foundation for Statistical Computing, Vienna, Austria).

## Results

In total, a McGRATH laryngoscope was used in 68 of 360 patients (18%). Tables 1 and 2 demonstrates the characteristics in patients who were used a McGRATH laryngoscope and who were used a Macintosh laryngoscope.

**Table 1:** Characteristics of patients with McGRATH laryngoscope and with Macintosh laryngoscope.

	McGRATH laryngoscope (n = 68)	Macintosh laryngoscope (n = 292)	P value	Adjusted P value
Sex (%)				
Male	26 (38)	114 (39)	1	0.49
Female	42 (62)	178 (61)		
ASA–PS (%)				
1	12 (17)	98 (34)	0.17	0.28
2	52 (77)	185 (63)		
3	4 (6)	9 (3)		
Age (year)	61 (52–73)	58 (46–71)	0.16	0.55
BMI (kg m-^2^)	24 (21–26)	23 (20–25)	0.10	0.51
History of hypertension (%)	24 (35)	95 (33)	0.67	0.32
Antihypertensive therapy (%)	22 (32)	81 (28)	0.76	0.34
Estimated glomerular filtration rate (mL min^-1^)	77 (19)	78 (23)	0.44	0.63
Diabetes mellitus (%)	13 (19)	31 (11)	0.06	0.46
Current smoker (%)	8 (12)	46 (16)	0.57	0.35
Systolic blood pressure on admission (mmHg)	129 (17)	127 (20)	0.46	0.61

Values are presented as mean (standard deviation), median (interquartile range), or number (proportions). ASA-PS, American Society of Anesthesiologists Physical Status.

**Table 2:** Anesthetic factors of patients with McGRATH laryngoscope and with Macintosh laryngoscope.

	McGRATH laryngoscope (n = 68)	Macintosh laryngoscope (n = 292)	P value	Adjusted P value
Propofol (mg kg-1)	1.5 (0–1.9)	1.4 (0.8–1.8)	0.87	0.36
Fentanyl (mcg kg-1)	2.9 (2.4–3.4)	2.7 (2.1–3.4)	0.43	0.56
Remifentanil (mcg kg-1 min-1)	0.06 (0–0.1)	0.05 (0–0.1)	0.3	0.76
Intubation operator (%)				
Resident	27 (40)	119 (41)	1	0.58
Senior resident	33 (48)	140 (48)		
Staff	8 (12)	33 (11)		
Intubation time (min)	8 (7–10)	8 (7–9)	0.46	0.37
Tube size (mm)	7 (7–7.5)	7 (7–8)	0.14	0.55

Values are presented as median (interquartile range), or number (proportions).

An increase in SBP of more than 20% after tracheal intubation occurred in 189 of 360 patients (53%). SBP increased more than 20% in 34 patients (50%) in the McGRATH group and 155 patients (53%) in the Macintosh group (Table 3). There was no significant difference in the time to measure blood pressure after intubation or the EtCO_2_ immediately after intubation between the two groups even after propensity adjustment using the IPW approach (Table 3).

**Table 3:** Results of blood pressure change, time to measure blood pressure, and EtCO_2_.

	McGRATH laryngoscope (n = 68)	Macintosh laryngoscope (n = 292)	P value	Adjusted MD or OR (95% CI)	Adjusted P value
Hypertension after intubation (%)	34 (50)	155 (54)	0.69	0.43 (0.19 to 0.96)	0.04
Change of SBP after intubation (mmHg)	24.1 (25.7)	29.0 (25.0)	0.24	-8.6 (-17.4 to 0.2)	0.06
Change of MBP after intubation (mmHg)	14.2 (21.7)	21.3 (20.0)	0.03	-9.5 (-17.4 to -1.6)	0.02
Change of DBP after intubation (mmHg)	12.6 (18.7)	17.6 (17.7)	0.09	-5.9 (-12.0 to 0.2)	0.06
Time to measure blood pressure after intubation (min)	1.7 (0.87)	1.6 (0.89)	0.55	0.1 (-0.2 to 0.4)	0.52
EtCO_2_ after intubation (mmHg)	39 (36–42)	38 (35–42)	0.46	1.1 (-2.1 to 4.4)	0.50

MD, mean difference; OR, odds ratio; CI, confidence interval; SBP, systolic blood pressure; Values are presented as mean (standard deviation), median (interquartile range), or number (proportions). The propensity scoring with inverse probability weighting adjusted OR and MD are presented for dichotomous and continuous data, respectively. MBP, mean blood pressure; DBP, diastolic blood pressure; EtCO_2_, end-tidal carbon dioxide.

Multicollinearity was found between history of hypertension and antihypertensive therapy in our data, and we consequently excluded antihypertensive therapy from the logistic regression for the propensity scoring. After propensity adjustment using the IPW approach, any of the parameters did not differ between the 2 groups (p values were <0.2 in all parameters as shown in the Tables 1 and 2). The odds ratio for the incidence of hypertension after the use of a McGRATH laryngoscope was 0.43 (95% CI, 0.19–0.96; P = 0.04) (Table 3). The C-index of the logistic model used to create the propensity score was 0.74 (95% CI, 0.66–0.82), and the P value of the Hosmer-Lemeshow test was 0.39. The odds ratio for the use of a McGRATH laryngoscope was 0.19 (95% CI, 0.08–0.42; P < 0.0001) and 0.50 (95% CI, 0.22–1.14; P = 0.10) when hypertension was defined as an increase in MBP or DBP >20%, respectively. The mean difference in the change of SBP after intubation between the two laryngoscopes was -8.6 mmHg (95% CI, -17.4 to 0.2; P = 0.06). The mean difference in the change of MBP or DBP after intubation is presented in Table 3.

## Discussion

Herein, we demonstrated that using a McGRATH laryngoscope may reduce the incidence of hypertension after tracheal intubation compared to a Macintosh laryngoscope.

Our finding is consistent with previous studies on other devices such as a light wand [8, 9], fiberoptic intubation [6], intubating Laryngeal Mask [8], and Airway Scope (PENTAX-AWS^®^; Pentax, Tokyo, Japan) [18, 19]. A Macintosh laryngoscope requires forced alignment of the oral and pharyngeal axes in order to view the glottis. This maneuver stimulates supraglottic regions and the oral tissue and induces the patient’s sympathetic response. It is considered to be a primary cause of an excessive hemodynamic response when the direct laryngoscopy technique is used. Because excessive stimulation of a supraglottic region during intubation can be avoided by using a light wand, fiberoptic intubation, intubating Laryngeal Mask, or Airway Scope, it is reasonable to speculate that these devices reduce the hemodynamic response. Similarly, we postulate that the McGRATH laryngoscope reduces the hemodynamic response to intubation, probably because it does not require the forcible alignment of the oral and pharyngeal axes in order to view the glottis.

When comparing a new airway device with the conventional Macintosh laryngoscope, proficiency in its use can be a confounding factor. The McGRATH laryngoscope was introduced to our institution approximately 1 year prior to the period covered by this study. At the time of the study, there was a McGRATH laryngoscope in every operating room and our department head recommended its use in routine cases. Moreover, it is very similar to the conventional Macintosh laryngoscope in design and technique for visualizing the larynx. Therefore, we believe it is accurate to assume that all anesthesiologists involved in this study were proficient in the use of the McGRATH laryngoscope. Furthermore, in less experienced hands, we would expect more stimulation and higher blood pressures, which could potentially obscure the beneficial effects of the McGRATH laryngoscope observed in this study.

The propensity score technique generally has the potential to derive a specific outcome from observational study data that is equal to the ability of a randomized controlled study to derive these outcomes. However, this technique could not remove the unmeasured or unknown confounders; thus, our hypothesis should be proven in future randomized controlled studies.

We recognize that our retrospective single center study has some limitations. First, we cannot reject the possibility that additional factors not included in our study contributed to the hemodynamic response during tracheal intubation. Second, we might have missed maximal or minimum SBP values, because SBP data were obtained retrospectively from the anesthetic records. As we already mentioned in the methods, in most of our cases, the interval of blood pressure measurement ranged from 2 to 5 minutes. In a future prospective study, a shorter measurement interval (e.g., every minute or less) should be used to obtain more accurate data for the hemodynamic response after tracheal intubation. Third, for the end measurement only the blood pressure was selected out of many possible hemodynamic response parameters. Many confounding factors can vary blood pressure, and our study design might not cover all of those factors. Fourth, the choice of patients in whom a McGRATH laryngoscope was used was left to the discretion of each anesthetist, and there are no established criteria or standards for its use at our institution. It is probable that there was a tendency towards using a McGRATH laryngoscope in cases in which intubation was predicted to be difficult, because visualization of the larynx is somewhat better with the McGRATH laryngoscope compared to the Macintosh. Some anesthetists may also have hesitated to use a McGRATH laryngoscope in routine cases because it requires a disposable blade and costs slightly more than a conventional Macintosh laryngoscope. However, it is likely that difficult intubations take more time, which would obscure the beneficial effects of the McGRATH laryngoscope on blood pressure after intubation observed in this study.

In conclusion, the use of a McGRATH laryngoscope may reduce the incidence of hypertension after tracheal intubation compared to the Macintosh laryngoscope. This hypothesis should be confirmed in future prospective randomized studies.

## Supporting Information

S1 Table Data of the patients inculded in this study. (DOCX)
