# Supplementary material for: Effect of the McGRATH MAC® Video Laryngoscope on Hemodynamic Response during Tracheal Intubation: A Retrospective Study
Source: PLoS One. 2016 May 12;11(5):e0155566. doi: 10.1371/journal.pone.0155566 (PMC4865033; doi:10.1371/journal.pone.0155566)
Supplement: S1 Table — (DOCX) [file pone.0155566.s001.docx]

S1 Table. Data of the patients included in this study.

Data were arranged in the following order from left; sex, age (yr), ASA-PS, BMI, history of HT, use of medication for HT, DM, current smoker, eGFR, dose of fentanyl, dose of remifentanil, dose of propofol, intubation operator code, use of McGrath, tube size, intubation time, baseline value of SBP, incidence.HT

1 70 1 16.6 0 0 0 0 61.6 5.0 0 1.98 1 0 7 8 136 0

1 60 2 23.9 1 1 0 0 111.5 3.7 0 1.10 1 0 7 NA 145 0

0 40 1 24.2 0 0 0 0 111 3.4 0 1.36 0 0 7 NA 110 1

1 44 1 17.0 0 0 0 0 91.3 2.3 0 NA 0 0 7 8 120 0

1 62 3 18.6 0 0 0 0 92 4.2 0 1.68 0 1 7 6 139 1

1 68 2 28.4 0 0 1 1 66.2 1.4 0 1.71 0 1 7 6 152 1

0 73 2 19.0 1 1 0 1 126.4 3.8 0 0.94 2 0 8 5 152 0

0 65 2 18.0 1 1 1 1 95.4 2.8 0 1.47 1 0 8 5 106 1

1 44 1 21.9 0 0 0 0 69.3 3.6 0 1.25 0 0 7 8 96 1

0 69 2 20.8 0 0 0 0 46.3 3.3 0 1.33 1 0 8 7 124 1

1 18 1 18.1 0 0 0 0 139.6 2.1 0 NA 0 1 6.5 11 118 0

1 49 2 29.6 1 1 0 0 60.7 1.6 0 2.22 0 0 7 8 140 1

0 59 3 25.5 1 1 1 0 57.1 2.5 0 2.26 1 1 8 7 107 1

1 74 2 24.3 0 0 0 0 80.2 3.7 0 0.92 0 0 7 9 134 0

0 80 1 27.2 0 0 0 0 40.7 1.4 0 2.30 2 1 7.5 8 134 1

0 49 2 24.3 1 0 0 0 59.6 2.7 0 2.01 1 1 8 9 140 1

0 72 3 28.1 1 1 0 0 33.5 4.2 0 0.99 1 0 8 8 137 0

1 42 2 27.9 0 0 0 0 78.6 2.2 0.2 NA 2 1 7 9 112 1

0 21 1 22.0 0 0 0 0 112.6 3.3 0 1.65 0 0 7.5 8 116 1

0 20 1 19.8 0 0 0 0 121.3 2.4 0.2 1.95 0 0 8 9 127 1

1 42 1 22.8 0 0 0 0 96.2 3.7 0 NA 1 0 7 4 128 1

1 38 1 24.9 0 0 0 0 77 3.0 0.1 1.79 0 1 7 7 130 0

1 43 1 21.9 0 0 0 0 78 3.4 0 1.36 0 0 7 9 112 0

0 58 1 26.0 0 0 0 1 75.9 2.7 0 0.80 1 0 8 5 116 1

1 57 1 21.7 0 0 0 0 65 1.8 0 NA 0 0 6 8 102 1

1 44 2 26.3 1 0 1 0 87.8 2.3 0 0.94 0 0 7 8 131 1

1 70 2 21.1 0 0 0 0 75.4 3.8 0 NA 1 1 7 9 106 0

1 62 2 22.3 0 0 0 0 69.1 3.8 0 0.95 0 0 7 9 158 1

1 57 1 25.2 0 0 0 59 1.5 0 1.49 0 1 7 5 108 1

0 72 2 22.7 0 0 1 0 103.2 2.7 0 1.79 0 0 8 8 152 1

1 70 2 19.7 0 0 1 0 53.3 4.5 0 NA 1 0 7 6 147 1

0 62 2 24.2 1 1 0 0 86.3 2.6 0 1.84 1 0 8 7 143 0

0 43 1 27.7 0 0 0 0 56 3.6 0 2.07 0 0 8 7 121 0

1 36 2 21.4 0 0 0 0 88 3.4 0.2 1.71 1 0 7 8 120 1

1 46 1 19.4 0 0 0 1 79 4.1 0 1.43 1 0 7 12 112 0

1 44 2 19.2 0 0 0 0 64.4 1.9 0.2 NA 0 0 7 13 113 0

1 61 1 24.7 0 0 0 0 77.2 2.8 0 2.28 0 0 7.5 10 148 1

1 80 2 21.3 0 0 0 0 64.2 3.3 0 0.87 0 1 7 11 118 0

1 70 2 18.6 0 0 0 0 75.4 4.7 0 1.63 1 0 7 6 129 1

0 47 1 24.0 0 0 0 1 112.4 1.4 0.3 2.45 0 0 8 5 140 1

1 47 2 20.9 0 0 0 0 80.1 4.1 0.1 2.86 0 1 6 9 128 1

1 42 1 19.3 0 0 0 1 93.1 1.9 0.4 2.88 0 0 7 11 103 0

1 76 2 22.7 1 1 1 NA 49.4 3.4 0 1.72 1 0 7 7 107 0

0 60 2 22.5 0 0 0 1 99.3 3.4 0 1.68 1 0 8 10 143 1

0 67 2 27.3 1 1 0 0 73.1 2.6 0 1.97 0 0 8 8 177 0

1 67 1 23.1 0 0 0 0 85.9 3.6 0 2.68 1 0 7 9 139 1

1 73 1 33.8 0 0 0 0 60.9 2.8 0 2.10 1 0 7 6 158 0

1 49 2 32.8 1 1 0 0 104 1.2 0 1.43 0 0 6.5 11 132 1

0 84 2 18.3 1 1 0 1 64.1 5.1 0 0.77 0 0 7.5 5 164 1

1 27 1 22.5 0 0 0 0 90.7 2.9 0 2.31 0 0 6.5 9 105 0

1 47 2 18.7 0 0 0 0 84.6 2.4 0.1 1.95 1 0 7 7 106 0

1 76 2 27.6 1 1 1 0 67.4 3.4 0 1.72 0 0 7 10 147 1

0 63 2 26.0 0 0 0 1 62.5 2.8 0 2.12 0 0 7.5 6 105 1

1 84 2 20.6 1 1 0 0 83.8 2.1 0 2.06 0 0 7 5 142 0

0 83 2 18.7 1 0 0 0 109.3 4.4 0 1.56 0 0 8 8 148 1

0 84 2 21.2 1 1 1 0 40 2.7 0.1 1.45 0 0 7.5 7 102 0

0 69 2 26.0 1 1 0 0 89.2 2.1 0 2.86 0 0 8 10 155 0

1 32 1 21.8 0 0 0 0 82.2 1.8 0 1.77 0 0 7 9 116 1

0 81 2 26.8 1 1 0 0 44.6 3.0 0 1.20 1 0 7.5 6 123 0

1 27 2 32.5 0 0 0 1 87.7 2.6 0.1 NA 0 1 7 7 111 0

0 72 2 14.0 1 1 1 0 118.8 5.5 0 0.82 2 0 8 6 114 0

1 66 2 28.1 1 1 0 0 92 2.3 0 0.94 0 0 7 10 128 0

1 65 2 21.5 1 1 0 0 69.3 3.8 0 NA 1 0 7 9 131 0

1 32 1 21.5 0 0 0 0 82.2 1.8 0 1.77 0 0 7 7 111 1

0 64 2 23.5 0 0 0 0 97.5 3.1 0 1.53 2 0 8 8 132 1

1 71 1 21.9 0 0 0 0 52.4 2.0 0 1.80 0 0 7 7 159 1

1 52 2 27.3 1 0 0 0 83.7 2.9 0 1.01 1 0 7 5 135 0

1 19 1 22.5 0 0 0 0 4.0 0 NA 2 0 6 9 85 1

1 73 2 19.6 1 1 1 0 78.9 2.0 0 1.02 0 1 7 10 125 1

1 79 2 20.2 1 1 0 0 43.3 1.1 0 1.60 1 0 7 7 147 1

0 65 1 20.9 0 0 0 1 64.9 3.4 0 1.72 1 0 8 8 102 1

1 66 1 22.7 0 0 0 0 76.7 3.8 0.1 1.33 0 1 6 10 139 0

1 80 2 22.1 0 0 1 0 64.2 2.0 0 0.98 0 0 7 9 136 1

0 74 2 22.6 0 0 1 0 69.2 3.3 0 1.00 0 0 8 7 161 0

0 39 2 25.8 0 0 0 0 80 1.4 0 1.94 1 1 7 6 123 0

1 79 2 21.9 1 1 0 0 83.7 4.2 0 1.25 1 0 7 8 144 1

0 77 2 20.0 1 0 0 0 77.5 2.3 0.1 1.49 0 0 8 7 157 0

1 45 1 24.9 0 0 0 0 109.8 3.2 0 1.90 1 0 7 6 115 0

1 78 2 24.6 1 1 0 0 75.9 1.8 0 0.89 0 0 7 8 142 1

1 69 2 24.1 1 1 0 0 90.8 3.6 0 NA 1 1 7 10 136 1

1 75 2 20.2 0 0 0 0 99.5 2.2 0 1.08 1 0 7 6 104 1

0 57 2 18.9 0 0 0 1 91.2 2.1 0 1.67 1 0 8 6 98 1

1 20 1 20.3 0 0 0 0 100.6 4.4 0 NA 1 0 7 8 118 0

1 81 2 19.8 0 0 0 0 102.6 2.2 0 1.51 0 0 7 8 121 1

1 54 2 32.8 1 1 0 0 74.4 2.9 0 NA 1 1 7 9 155 1

0 77 2 23.8 0 0 1 0 50.7 3.1 0 1.56 1 1 8 9 145 1

0 74 3 22.9 0 0 1 0 74 1.8 0 1.06 0 0 7.5 8 138 1

1 60 2 23.1 0 0 1 0 86.9 3.5 0 1.75 1 1 7 10 140 1

0 21 2 31.0 0 0 0 0 81.9 2.1 0 NA 1 1 8 8 142 1

1 70 1 17.5 0 0 0 0 63.6 4.8 0 1.43 1 0 7 8 100 1

1 40 2 23.1 0 0 0 0 90.3 1.6 0.2 NA 1 0 7 8 126 0

1 43 1 20.7 0 0 0 0 69.8 2.7 0 1.64 1 0 7 5 100 0

0 62 1 18.7 0 0 0 0 72.8 2.0 0 1.76 0 0 8 8 114 1

0 77 2 23.5 1 1 0 0 50.7 3.2 0 1.60 0 0 8 8 122 0

1 70 1 31.5 0 0 0 0 61.6 1.4 0.15 1.45 0 0 7 7 141 1

0 59 2 24.0 0 0 0 0 88.9 2.6 0 1.30 1 0 8 9 117 1

1 18 1 21.4 0 0 0 0 1.6 0.15 NA 0 0 7 9 118 1

1 77 2 28.2 1 1 1 0 40.8 3.1 0 0.92 1 0 7 6 175 1

1 53 2 23.6 0 0 0 0 95.8 2.7 0 1.99 0 0 7 9 132 1

0 73 2 23.2 1 1 0 0 69.4 1.5 0 1.19 0 0 8 8 150 1

1 66 2 22.8 0 0 0 0 79.7 3.9 0 NA 1 1 7 10 123 0

0 54 2 30.9 0 0 0 1 71 2.2 0 1.62 0 0 8 8 129 1

0 72 2 22.9 0 0 0 0 59.4 1.7 0 0.87 1 0 8 8 123 0

1 48 2 22.6 0 0 0 0 66.6 3.4 0 2.05 1 0 7 10 121 0

0 36 2 22.9 0 0 1 0 87.3 1.9 0 0.75 1 1 8 6 118 0

0 64 2 25.9 1 1 0 0 59.5 2.9 0 0.74 1 0 8 7 127 1

1 75 2 22.6 1 1 0 0 48.4 3.7 0 0.93 0 0 7 9 110 0

1 34 1 32.1 0 0 0 0 94.6 2.5 0 1.48 0 0 7 10 126 0

1 77 2 19.6 1 1 0 0 14.7 4.4 0 1.09 0 0 7 6 170 1

1 55 1 22.5 0 0 0 1 70.3 1.9 0 NA 1 0 6.5 5 132 0

0 65 2 26.9 1 1 0 0 59.2 1.3 0 1.30 1 0 7 9 111 1

1 42 1 19.3 0 0 0 0 89 4.3 0 NA 1 1 7 9 118 1

1 78 2 20.8 1 1 0 0 94.1 4.8 0 0.71 1 0 7 6 130 0

1 50 2 23.9 0 0 0 0 95.4 3.1 0 2.80 0 0 7 6 124 1

1 42 2 20.3 0 0 0 0 94.3 3.3 0.2 1.56 0 0 7 11 113 1

0 70 2 26.2 1 0 0 0 37.6 2.1 0 1.69 1 0 8 7 170 1

0 70 2 17.8 0 0 0 1 73.2 3.8 0 1.69 0 0 8 9 109 1

1 78 2 22.9 1 1 0 0 79 1.9 0 0.94 1 0 7 7 174 1

0 42 2 28.9 0 0 0 1 88.3 2.6 0 1.56 1 0 8 6 119 1

1 66 2 24.5 0 0 0 0 75.3 1.6 0 1.31 0 0 7 11 124 1

0 82 2 25.6 0 0 1 1 63 2.6 0 1.20 0 1 8 12 128 0

1 57 1 19.1 0 0 0 0 80 2.1 0 NA 2 0 7 8 125 1

1 79 2 34.2 1 1 1 0 51.5 1.4 0 1.14 2 1 7 8 128 1

0 60 1 22.5 0 0 0 1 80.9 1.5 0 2.53 1 0 8 6 127 1

0 77 2 25.3 0 0 0 0 52.9 3.2 0 1.12 2 0 8 8 138 1

1 52 2 29.5 0 0 0 0 68.5 2.9 0.2 1.43 1 0 7 5 126 0

1 70 1 21.4 0 0 0 0 65.6 3.0 0 1.01 1 0 7 9 109 0

0 66 2 29.8 1 1 0 1 91.8 2.4 0 1.46 1 1 7.5 6 140 0

1 47 2 18.8 0 0 0 1 73.6 4.3 0 NA 0 0 7 10 93 0

1 54 2 31.4 1 1 1 0 89.5 2.6 0 NA 1 1 7 7 138 1

1 58 1 25.5 0 0 0 0 95.4 2.3 0 1.55 1 0 7 9 149 1

0 22 2 28.4 0 0 0 1 103.4 2.5 0.1 1.85 0 0 8 8 145 0

1 48 1 24.1 0 0 0 1 76.9 3.3 0 1.64 0 0 7 8 108 1

1 60 2 18.1 0 0 0 0 58 2.1 0 1.28 2 0 7 5 104 1

1 77 2 24.1 1 1 0 0 35 1.7 0.2 1.21 1 0 7 9 112 0

0 74 2 29.3 1 1 1 1 48.9 2.5 0 1.49 1 0 7.5 16 146 0

0 67 1 22.7 0 0 0 0 77.2 2.8 0 1.42 1 0 8 11 119 1

1 38 3 21.8 0 0 0 0 103.2 2.0 0.2 NA 1 0 7 7 133 0

0 83 2 18.1 1 1 1 0 11.6 4.0 0 0.60 1 0 8 10 220 1

0 87 2 24.1 1 1 0 1 61.2 1.5 0.1 0.59 2 0 7.5 8 115 0

1 81 2 21.0 0 0 0 0 79.7 2.5 0.1 NA 1 0 6.5 10 132 1

1 40 2 29.3 0 0 0 0 93.8 2.6 0 1.97 1 0 7 8 131 0

1 72 1 24.0 0 0 0 0 55.9 1.7 0 NA 2 1 7 9 126 1

1 27 1 20.1 0 0 0 0 76.3 2.7 0.2 2.14 1 0 6 8 125 0

1 43 1 21.2 0 0 0 0 104.7 4.1 0 3.06 0 0 7 5 100 1

1 71 1 26.0 0 0 0 0 71.2 1.7 0 0.83 0 0 7 9 138 1

1 66 3 22.3 0 0 0 0 74 3.5 0 NA 1 1 7.5 5 126 0

0 21 1 20.8 0 0 0 1 94.3 3.7 0 NA 1 0 8 6 102 0

0 74 2 21.1 1 0 0 0 52.4 3.6 0 1.45 1 0 8 9 148 1

0 67 2 26.7 1 1 1 0 31.9 2.8 0 1.69 1 0 8 4 122 0

0 64 2 22.5 0 0 1 0 148 3.0 0 1.20 1 0 8 9 115 0

0 72 2 20.3 0 0 1 0 64.6 1.9 0.2 NA 1 0 8 8 118 1

0 36 2 25.9 0 0 0 0 78.8 2.7 0 2.03 1 0 7.5 8 124 0

1 41 1 20.5 0 0 0 0 105.4 3.1 0 NA 2 0 7 6 115 0

1 64 2 18.5 0 0 0 0 66.3 4.2 0 NA 2 1 7 6 113 0

1 40 1 20.9 0 0 0 0 101.7 2.1 0.4 2.55 2 0 7 9 103 0

0 77 2 22.9 1 1 0 0 61.1 2.3 0 2.03 1 0 8 7 155 0

0 62 2 26.8 1 0 0 1 72.8 1.9 0 1.55 1 0 8 12 155 1

1 83 2 19.9 0 0 0 0 84.3 4.1 0.15 NA 1 0 7 7 146 0

0 54 2 22.4 0 0 0 1 43.1 3.3 0 2.46 0 1 7 12 126 0

0 70 2 24.9 1 1 0 0 117.7 1.7 0 2.03 0 0 8 10 137 1

0 49 1 29.1 0 0 0 0 70 2.4 0.1 1.79 0 0 8 7 131 1

1 72 2 25.4 1 1 0 1 45.5 2.6 0 1.72 0 0 7 9 140 1

0 68 2 27.7 1 1 0 0 66.8 1.2 0 1.22 1 0 8 7 141 1

0 57 1 25.1 0 0 0 0 74.5 1.4 0.25 1.69 0 0 8 8 111 0

1 49 2 20.1 0 0 0 0 98 3.1 0 2.07 0 0 7 7 128 0

0 38 2 25.0 0 0 0 0 64.7 2.9 0 NA 0 0 8 9 130 0

1 51 1 24.4 0 0 0 0 84.2 2.4 0 1.60 1 0 7 9 102 0

1 46 2 18.7 1 1 0 0 80.6 4.6 0 1.85 2 0 7 7 141 0

1 66 3 21.9 0 0 0 1 21.6 2.0 0 0.80 0 0 7 6 100 0

1 21 2 20.1 0 0 0 0 99.2 2.7 0.25 NA 0 0 7 11 92 0

0 80 2 24.2 1 1 0 0 61.9 1.4 0 2.32 1 1 8 12 113 1

0 40 1 29.4 0 0 0 1 77 2.2 0 1.32 1 0 7.5 9 136 1

0 46 2 21.6 0 0 0 0 88.6 2.9 0 2.02 1 0 8 4 121 0

1 26 1 19.0 0 0 0 0 128.6 0.0 0.2 NA 2 0 7 9 98 0

0 68 2 22.3 1 1 0 0 92 3.3 0 1.63 1 0 8 9 155 0

1 28 2 20.1 0 0 0 0 92.9 1.8 0 2.19 2 0 7 6 98 1

1 46 2 26.2 0 0 0 0 79.2 3.6 0 NA 1 0 7 9 124 1

1 63 2 19.9 1 0 1 0 80.7 4.0 0 2.02 0 0 7 5 120 0

1 42 1 25.0 0 0 0 102.4 1.6 0 NA 2 0 7 98 0

0 72 2 17.7 1 1 1 0 118.8 3.2 0 1.05 0 1 7.5 7 89 0

1 59 1 20.2 0 0 0 0 82.3 4.0 0 1.98 0 0 7 7 122 0

1 43 2 40.9 1 1 1 0 88.4 2.1 0.15 0.74 2 0 7 7 133 0

1 46 2 25.4 0 0 0 90.1 2.9 0 1.90 0 1 7.5 9 138 0

1 22 1 22.7 0 0 0 0 96.2 2.5 0.15 2.04 0 0 6.5 5 105 0

0 71 3 22.5 0 0 1 5.4 2.6 0.1 0.86 0 0 8 6 126 1

0 76 2 22.6 1 1 0 0 59.9 3.1 0.2 1.86 0 1 8 5 138 1

1 64 2 39.3 0 0 0 0 69.6 2.1 0 0.74 0 0 7 5 149 1

1 57 2 29.7 0 0 0 0 122.4 1.4 0.1 NA 1 0 7 10 110 0

0 74 2 23.3 1 1 1 0 80.8 3.0 0 1.49 0 0 8 4 128 1

1 61 2 20.1 1 1 0 0 92 3.3 0 NA 1 0 7 6 157 0

1 43 2 33.4 1 0 0 0 2.6 0.6 0.2 1.14 1 0 7 10 133 1

1 63 2 25.5 0 0 0 1 58.9 1.7 0 NA 1 0 7 8 124 0

1 80 2 18.4 1 1 1 0 75.4 4.2 0 2.25 0 1 6.5 8 141 1

1 33 2 18.2 0 0 0 0 107.5 1.1 0.4 2.25 1 0 6.5 6 89 0

0 65 2 24.4 0 0 1 0 80.2 2.5 0.15 1.68 1 0 8 11 132 1

1 40 2 16.0 0 0 0 0 108.5 2.6 0.3 2.34 1 0 6.5 8 145 0

0 71 2 23.8 1 1 0 0 54.7 3.4 0 3.03 1 0 8 6 165 0

0 69 2 27.0 0 0 0 0 85 3.8 0.15 NA 2 1 8 6 112 0

0 55 1 23.7 0 0 0 0 86.7 2.1 0 1.53 2 0 8 8 138 0

1 52 1 22.0 0 0 0 1 96.4 2.8 0.15 2.77 0 1 6.5 10 154 0

0 69 2 23.4 0 0 0 1 115 2.0 0.25 1.56 1 0 7.5 7 144 0

1 44 1 18.8 0 0 0 0 51.2 4.5 0 NA 0 0 7 6 118 0

1 67 2 26.5 0 0 0 0 84.2 3.2 0.2 1.59 0 1 7 8 180 0

1 51 2 30.8 0 0 0 1 94.9 2.8 0.5 NA 0 1 7 10 136 0

0 63 3 19.1 0 0 0 0 5.1 3.0 0.1 1.58 1 0 8 4 135 0

1 61 2 18.8 0 0 0 0 81.5 4.6 0.05 1.84 0 0 7 5 100 1

1 86 2 27.8 1 1 0 0 44.8 2.5 0.25 1.50 1 0 7 10 146 0

1 64 2 22.3 0 0 0 0 57 3.2 0 NA 1 0 6.5 8 127 1

0 75 2 19.9 1 1 0 0 66.4 3.4 0 1.72 0 0 8 8 136 0

1 73 1 24.0 0 0 0 0 89 1.8 0.15 1.25 0 0 7 9 162 1

0 78 2 23.7 1 1 0 0 78.4 1.6 0 1.65 0 0 7 7 150 1

1 59 2 19.9 0 0 0 1 95 4.1 0 NA 0 0 7 16 108 0

0 61 2 23.4 0 0 0 0 69.4 2.4 0 1.27 1 0 8 5 138 1

1 60 2 26.6 1 1 0 0 88.7 3.5 0 2.65 2 0 7.5 9 132 0

1 73 1 21.8 0 0 0 0 65.9 2.6 0.1 NA 0 0 7 9 152 1

0 77 1 24.1 0 0 0 1 54 3.1 0 1.41 0 0 8 8 133 1

1 41 1 23.1 0 0 0 0 124.3 1.8 0 1.83 1 0 7 10 93 0

0 45 1 15.4 0 0 0 0 73.9 0.0 0.25 2.22 1 0 8 7 128 0

1 46 1 25.1 0 0 0 0 90.1 2.4 0.3 NA 0 0 7 9 124 0

0 87 2 22.7 1 1 0 0 57 2.6 0 0.86 1 0 8 8 135 1

1 68 2 21.7 1 1 0 0 50.4 2.9 0 1.56 1 0 7 7 109 1

1 22 1 21.3 0 0 0 0 91.2 2.8 0.1 NA 0 0 7 9 120 0

1 82 2 24.4 0 0 0 0 46 3.5 0 NA 2 0 7 5 120 0

1 56 1 22.4 0 0 0 0 67.8 3.8 0 1.90 1 0 7 7 110 0

1 83 2 23.6 1 0 0 0 56.9 2.0 0 1.02 1 0 7 5 141 0

1 66 2 21.1 0 0 0 0 88.1 1.9 0.1 1.95 1 1 7 9 140 1

0 73 2 24.9 1 1 1 1 51 1.5 0 1.47 1 0 8 6 155 1

1 49 2 19.4 0 0 0 0 104.7 4.2 0 2.30 2 0 7 16 100 1

1 46 2 34.9 0 0 0 1 118 2.8 0 1.02 2 0 7 6 138 0

0 79 2 25.7 0 0 0 0 56 2.9 0 1.01 1 0 8 7 144 1

0 66 2 22.4 1 1 0 0 83.5 2.5 0.2 1.68 0 1 7.5 10 134 0

0 70 2 23.1 0 0 0 0 43 3.3 0 2.50 1 0 8 16 136 1

0 59 1 18.7 0 0 0 0 82 3.9 0.1 NA 1 0 7.5 7 92 1

1 58 2 14.0 0 0 0 0 67.1 3.0 0 1.81 1 0 7 7 126 1

0 80 2 26.0 1 1 0 0 68.5 2.4 0.15 NA 0 1 7.5 15 120 1

1 46 2 16.8 0 0 0 0 61.8 2.2 0 1.77 1 0 7 10 119 0

1 55 2 19.8 0 0 0 0 94 2.1 0 1.45 1 0 7 6 109 1

1 74 2 23.8 1 1 0 0 63.5 2.7 0.15 NA 1 0 7 7 112 1

1 64 2 21.3 1 1 0 0 87 3.1 0 1.65 1 1 7.5 9 145 1

1 63 2 23.3 1 1 0 0 99.7 1.8 0.1 NA 1 0 7 6 133 0

1 43 1 19.7 0 0 0 0 88.4 4.3 0 3.26 1 0 7 9 99 0

0 60 2 23.7 1 0 0 1 88.5 2.3 0 1.24 1 0 8 11 157 1

0 70 3 24.2 1 1 1 0 43 1.8 0.5 NA 0 1 6.5 6 120 0

1 60 2 18.0 1 1 0 0 63.4 4.6 0 NA 1 0 7 7 161 1

1 64 2 18.4 0 0 0 0 61.3 4.5 0 NA 0 0 7 8 123 1

0 81 2 19.1 0 0 1 0 77.6 3.7 0 2.02 0 0 8 8 122 1

1 49 1 18.8 0 0 0 0 66.2 4.2 0 1.66 0 0 7 8 112 0

0 62 1 19.7 0 0 0 1 81.3 3.1 0.1 1.57 2 0 7.5 6 119 1

1 60 2 25.2 1 1 1 0 50.3 1.5 0.2 NA 0 0 7 12 128 0

1 45 1 26.0 0 0 0 0 88.9 1.9 0.2 NA 0 0 7 9 135 0

1 71 2 22.8 1 1 0 86.3 2.9 0.2 NA 1 0 7 9 132 0

1 52 1 17.6 0 0 0 0 82.2 4.4 0 NA 2 0 7 14 110 1

0 28 1 20.3 0 0 0 0 92.6 3.7 0 1.85 1 0 8 8 104 0

0 74 2 24.8 1 1 0 0 44.2 3.0 0 2.40 2 0 7.5 6 109 0

1 33 2 18.4 0 0 0 0 49.3 4.5 0.25 NA 1 0 7 6 150 0

1 49 1 18.8 0 0 0 0 90.2 3.4 0 1.80 0 0 7 10 115 0

0 76 2 20.8 1 1 1 0 58.1 2.5 0 1.68 2 1 8 8 145 1

1 65 2 18.2 0 0 0 0 77.1 4.8 0 2.14 1 0 7 5 98 0

1 83 2 19.8 0 0 0 0 84.3 4.2 0.15 NA 1 0 7 11 146 0

0 62 2 26.8 1 0 0 1 72.8 1.9 0.2 1.55 1 0 8 12 155 1

0 77 2 23.0 1 1 0 0 61.8 2.4 0 2.05 1 0 8 7 155 0

1 40 1 20.6 0 0 0 0 111 2.2 0.4 2.59 2 0 7 10 103 0

1 41 1 20.4 0 0 0 0 105.4 3.1 0 NA 2 0 7 6 115 0

0 69 1 23.7 0 0 0 1 80 1.5 0.25 1.80 0 0 7.5 7 131 0

0 67 2 28.1 1 1 0 0 79.5 1.2 0 1.22 0 0 8 8 141 1

0 64 2 23.1 0 0 0 0 85.5 3.2 0 2.38 0 1 7 14 126 0

1 72 2 25.6 1 1 0 0 45.5 2.6 0 1.21 0 0 7 10 140 1

0 88 2 17.0 0 0 0 41.7 4.7 0 1.41 2 0 7.5 4 129 0

1 60 2 25.9 0 0 0 0 69.7 1.7 0 1.35 2 0 7 3 94 0

0 48 1 28.6 0 0 0 0 70 2.4 0.1 1.81 0 0 8 7 131 1

0 70 2 24.9 1 1 0 0 117.7 1.7 0 2.03 0 0 8 11 137 1

0 54 1 22.3 0 0 0 0 43.1 3.3 0 2.47 0 1 7 18 126 0

1 67 2 22.3 1 1 0 0 105.3 2.7 0 1.82 0 0 7 9 107 0

1 44 2 18.3 0 0 0 0 50.6 3.3 0 NA 1 0 7 5 99 1

1 75 2 22.5 1 1 0 0 55.3 2.6 0 1.58 1 1 7 8 92 0

1 54 2 38.1 0 0 0 0 82.8 2.0 0 1.00 0 0 7 11 128 0

1 66 1 26.8 0 0 0 0 66.8 1.7 0 1.21 1 0 7 8 122 1

1 71 2 21.5 0 0 0 0 111.9 6.8 0 NA 0 0 7 8 127 0

0 56 1 25.8 0 0 0 0 93.2 2.6 0.2 NA 1 1 8 9 138 1

1 53 2 20.3 0 0 0 0 48.6 1.9 0 1.90 1 1 7 8 113 1

1 43 3 24.1 1 1 0 0 95.6 3.1 0 1.09 0 0 7 11 119 1

1 50 3 26.5 0 0 0 0 83.1 2.9 0 1.46 0 0 7 7 124 0

0 59 2 21.6 0 0 0 0 69.2 1.5 0.1 1.21 0 0 8 9 96 1

1 57 2 27.7 1 1 0 1 78.6 1.6 0.2 2.34 1 0 6 9 132 1

1 47 2 26.1 0 0 0 0 97.1 3.2 0 NA 0 0 7 6 109 1

0 66 2 26.2 1 1 0 0 83.5 2.9 0 NA 1 0 8 10 150 1

1 43 2 28.1 1 1 0 0 64.8 3.3 0 NA 0 0 7 13 156 1

1 43 2 17.7 0 0 0 0 91 4.8 0.2 2.38 1 1 7 4 107 0

1 72 2 28.3 1 1 0 0 69.6 3.8 0 1.32 0 0 6.5 12 155 1

1 27 1 18.7 0 0 0 1 86.3 3.2 0.25 NA 1 0 7 16 106 0

1 81 1 23.0 0 0 0 0 92.8 4.0 0.25 1.00 1 1 7 6 134 1

1 68 2 18.0 1 0 0 0 110.3 1.2 0.2 NA 1 0 7 7 126 0

0 76 2 28.5 0 0 0 0 92.8 3.2 0 NA 2 1 8 9 110 1

0 66 1 22.6 0 0 0 0 90.3 2.1 0 1.38 0 0 7.5 9 129 0

1 51 2 24.0 1 1 0 0 71 2.9 0.1 1.52 1 1 7 8 144 0

1 70 2 16.0 1 1 0 0 75.4 4.2 0.15 2.25 1 0 6.5 7 136 1

0 74 2 21.4 1 1 0 0 65.7 2.4 0 2.43 1 1 7 7 118 1

1 47 1 28.0 0 0 0 0 95.7 3.2 0 0.79 1 0 7 10 112 0

1 70 1 24.8 0 0 0 0 65.6 3.1 0.2 1.23 1 1 7 10 114 0

0 64 2 27.1 1 1 0 0 73.4 1.3 0 2.14 2 0 7.5 6 106 1

1 26 1 18.0 0 0 0 0 111.7 3.3 0 NA 0 0 7 9 102 0

1 73 1 20.0 0 0 0 0 65.9 4.2 0.2 2.08 1 0 7 7 146 1

1 68 2 23.3 1 1 0 0 64.4 2.0 0 1.84 1 1 7 6 169 1

1 60 1 22.8 0 0 0 0 92.9 3.8 0 1.54 0 0 7.5 4 106 0

0 67 2 20.2 0 0 1 0 74.1 3.8 0 0.94 1 0 7.5 9 140 1

1 66 1 21.1 0 0 0 0 60.1 2.1 0 1.46 1 0 7 8 142 1

0 73 2 27.3 1 1 1 1 82.4 2.6 0 0.91 1 0 7.5 13 131 1

1 51 2 25.8 0 0 0 0 89.2 1.6 0.2 1.61 1 0 7 10 168 0

0 67 2 22.0 0 0 0 0 77 3.1 0.25 1.85 1 1 8 7 146 0

0 57 2 25.5 1 1 0 0 66.2 3.0 0 1.49 0 0 8 8 120 0

0 49 2 19.0 1 0 0 1 125 3.8 0 1.51 1 0 8 10 136 1

1 80 2 29.8 0 0 0 0 59.3 2.5 0 1.15 0 0 7 9 139 1

1 64 2 30.1 1 1 0 0 112.3 2.6 0 1.02 2 0 7 10 115 0

0 54 1 24.5 0 0 0 0 89.9 2.5 0.05 1.68 1 0 8 8 130 1

1 40 2 17.7 1 1 0 0 119.1 3.5 0 0.70 1 1 7 4 130 0

1 71 2 19.6 1 1 0 0 68.7 2.3 0 1.14 0 0 7 9 151 1

0 73 2 25.1 1 1 0 0 74.3 1.7 0.3 NA 1 0 8 7 124 0

0 68 1 21.0 0 0 0 0 62.6 3.5 0 1.90 0 0 8 9 112 1

1 67 2 28.8 1 1 0 0 88 2.8 0 1.69 1 1 7 6 151 0

1 72 2 27.8 1 1 1 0 105.8 3.2 0 1.13 1 0 7 6 168 1

0 69 2 23.6 0 0 1 0 60.2 3.0 0 1.50 1 0 8 6 140 1

1 38 2 17.1 0 0 0 0 78.2 5.0 0 2.50 1 0 7 6 108 1

1 52 1 21.8 0 0 0 85.3 3.8 0 1.15 1 0 6.5 7 98 1

1 47 2 24.8 0 0 0 0 114 2.4 0 0.97 0 0 7 12 145 1

1 45 2 33.3 1 1 0 0 74.5 2.7 0.1 1.64 0 0 7 8 126 1

0 66 2 25.3 1 1 0 0 58.3 1.4 0 1.35 1 0 8 8 152 1

0 79 2 18.1 0 0 1 0 90.5 2.3 0 1.14 0 0 8 9 133 1

0 24 2 30.0 0 0 0 0 100 1.6 0 2.44 1 0 7 8 119 1

1 37 2 26.1 0 0 0 0 98.6 3.0 0.1 NA 0 1 7 8 156 0

1 26 1 20.3 0 0 0 0 102 3.8 0.2 NA 1 0 7 10 103 0

0 41 1 22.6 0 0 0 1 92.9 2.9 0.1 2.57 1 0 7.5 10 125 0

1 75 2 25.7 0 0 0 0 58 2.9 0 0.72 1 1 7 6 121 1

0 42 1 28.3 0 0 0 0 85.3 2.5 0 1.50 2 0 8 10 117 1

0 36 1 22.9 0 0 0 1 95.8 3.2 0 NA 0 0 8 7 130 0

1 55 2 20.4 0 0 0 0 87.3 3.4 0.2 2.54 0 1 7 9 111 0

1 44 2 17.4 0 0 0 0 93.1 2.3 0 2.27 0 0 7 8 160 0

1 28 2 32.5 0 0 0 0 114 2.6 0 1.84 2 0 7 8 117 0

1 72 2 20.9 0 0 0 0 69.6 4.1 0 1.43 1 0 7 9 91 1

1 64 2 21.9 0 0 0 0 59.5 1.9 0 1.30 0 0 7 9 108 0

0 32 1 19.1 0 0 0 118 3.6 0.25 NA 2 0 8 6 129 0

1 78 2 19.1 0 0 0 0 63.6 2.2 0 1.79 1 0 7 5 124 1

1 26 1 24.5 0 0 0 0 112.7 1.9 0 NA 1 0 7 10 128 0

0 77 2 22.4 0 0 0 115.5 3.8 0 0.94 1 1 7 7 111 1

0 53 1 22.6 0 0 0 0 96.7 2.8 0 1.68 2 0 8 9 115 1

1 55 1 25.6 0 0 0 0 74.4 3.2 0 NA 1 0 7 9 146 1

1 58 2 16.0 1 1 0 0 67.1 2.0 0.2 NA 1 0 7 8 175 0

1 76 2 21.6 1 1 0 1 30.3 2.9 0.1 1.17 0 0 7 6 114 1

1 75 3 17.6 0 0 0 0 53.7 2.5 0.15 NA 0 0 7 12 151 1

1 32 1 20.9 0 0 0 1 77.8 4.1 0 3.67 0 0 7 12 110 1

1 49 1 24.3 0 0 0 0 64.3 3.3 0.2 NA 1 0 7 9 129 0

0 53 1 20.9 0 0 0 1 94.7 0.0 1 1.49 0 0 7 14 98 1

0 63 1 22.0 0 0 0 1 78.6 3.2 0 1.61 1 0 8 9 97 1

0 75 2 24.8 0 0 1 0 84.7 2.9 0 1.00 0 0 7.5 10 146 1

1 77 2 21.9 1 1 0 0 61.8 3.6 0 0.89 1 0 7 10 141 0

1 45 2 20.1 0 0 0 0 90.7 3.8 0.1 NA 0 0 7 9 109 0

1 56 2 28.6 0 0 0 1 51.9 2.6 0 1.71 0 1 7 12 118 0

0 78 2 19.0 1 0 0 0 79.6 4.2 0 1.25 1 1 7.5 7 162 0

1 39 1 23.0 0 0 0 0 93.4 2.5 0 NA 1 0 7 10 115 0

0 65 2 23.7 0 0 0 0 61.7 3.2 0.1 1.59 1 0 7.5 10 126 0
